# Daytime sleepiness and health-related quality of life in patients with childhood-onset craniopharyngioma

**DOI:** 10.1038/s41598-025-94384-5

**Published:** 2025-03-19

**Authors:** Laura Verena Mann-Markutzyk, Julia Beckhaus, Jale Özyurt, Aylin Mehren, Carsten Friedrich, Hermann L. Müller

**Affiliations:** 1 Department of Pediatrics and Pediatric Hematology/Oncology, University Children’s Hospital, Carl von Ossietzky Universität Oldenburg, Klinikum Oldenburg AöR, Rahel-Straus-Strasse 10, 26133 Oldenburg, Germany; 2 Biological Psychology Lab, Department of Psychology, School of Medicine and Health Sciences, Carl von Ossietzky Universität Oldenburg School IV, Oldenburg, Germany; 3 Department of Psychiatry and Psychotherapy, University of Bonn, Bonn, Germany; 4 Department of Cognitive Neuroscience, Donders Institute for Brain, Cognition and Behaviour, Radboud University Medical Centre, Nijmegen, The Netherlands

**Keywords:** Craniopharyngioma, Fatigue, Daytime sleepiness, Circadian rhythm, Hypothalamus, Quality of life, Endocrinology, Oncology

## Abstract

Overall survival rates after craniopharyngioma (CP) are high (92%), but frequently quality of life (QoL) is impaired in patients with CP involving hypothalamic structures. Tumour- and/or treatment-related hypothalamic lesions may result in disturbances of circadian rhythms including increased daytime sleepiness. We investigated the relationship between health-related QoL and daytime sleepiness in patients with childhood-onset CP. After a median follow-up of 10 years (range: 1–39), 119 CP patients (63 female), who were recruited 2000–2022 in the KRANIOPHARYNGEOM 2000/2007 and KRANIOPHARYNGEOM Registry 2019 trials, were assessed for daytime sleepiness using the Epworth Sleepiness Scale (ESS) and for QoL by EORTC QLQ-C30 questionnaire. CP patients with increased daytime sleepiness (ESS score > 10, *n* = 34) had worse self-assessment of QoL (*p* = 0.003), when compared to CP patients with normal ESS scores (*n* = 85). Increased daytime sleepiness was negatively correlated with QoL (*r*=-0.395; *p* < 0.001). Surgical hypothalamic lesions, detectable after surgical intervention in 92.9% of the reference-assessed patients, were associated with significantly higher ESS scores, whereas such impact could not be observed for presurgical hypothalamic involvement of the CP (72.4% of the reference-assessed patients). Compared to patients with an ESS score in the normal range, patients with increased daytime sleepiness suffered from impaired QoL in all functional scales and the global QoL scale of the EORTC QLQ-C30. As increased daytime sleepiness plays an important role for QoL in survivors of CP, hypothalamus-sparing surgical treatment strategies should be considered as state of the art in patients with CP for prevention of increased daytime sleepiness.

*Clinical trial registration * NCT01272622; NCT04158284, NCT00258453.

## Introduction

Craniopharyngioma (CP) is a rare, histologically benign tumour that shows no morphological signs of malignancy and originates from embryonic, ectodermal remnants of Rathke’s pouch^1^. The incidence of CP is 0.5 to 2.0 cases per million people per year – 30 to 50% of these are children and adolescents. Accordingly, 1.2–4.6% of all intracranial tumours in this age group can be attributed to CP^2–4^.

The anatomical location of the tumour requires individual treatment approaches. There is currently no standardised treatment for CP^5,6^. Several treatment methods such as surgical removal (complete resection vs. incomplete resection and radiotherapy)^5^, various access routes, radiotherapy, adjuvant or targeted therapies are available^7^. Ideally, each therapy represents an individualised case-by-case decision by an experienced multidisciplinary team^5^.

The question of health-related quality of life (QoL) is becoming increasingly important when making treatment decisions. CP patients with hypothalamic involvement frequently experience severe weight gain^8–10^, fatigue^11^, daytime sleepiness and secondary narcolepsy^12–19^, vascular complications^20–22^, dyspnoea, diarrhoea, reduced motivation and reduced/impaired physical activity^23^ and psychosocial development^24,25^. Neuroendocrine complications such as circadian rhythm disturbances can occur as part of a hypothalamic syndrome^26–28^. As a result, there is a massive reduction in QoL, which depends not only on the disease itself, but also on the type of therapy^29^. Eveslage et al.^30^ showed that QoL was reduced after a complete resection compared to an incomplete resection. After further treatment, such as radiotherapy, QoL of CP patients can also be impaired^31–33^. Excessive daytime sleepiness is a common symptom of hypothalamic syndrome that affects QoL and activities of daily living^13,34^.

This study aimed to analyse a possible association between the daytime sleepiness and QoL after CP therapy. In addition, we aimed to identify risk factors for increased daytime sleepiness and potential strategies for prevention.

## Patients and methods

### Patients

**Fig. 1:**
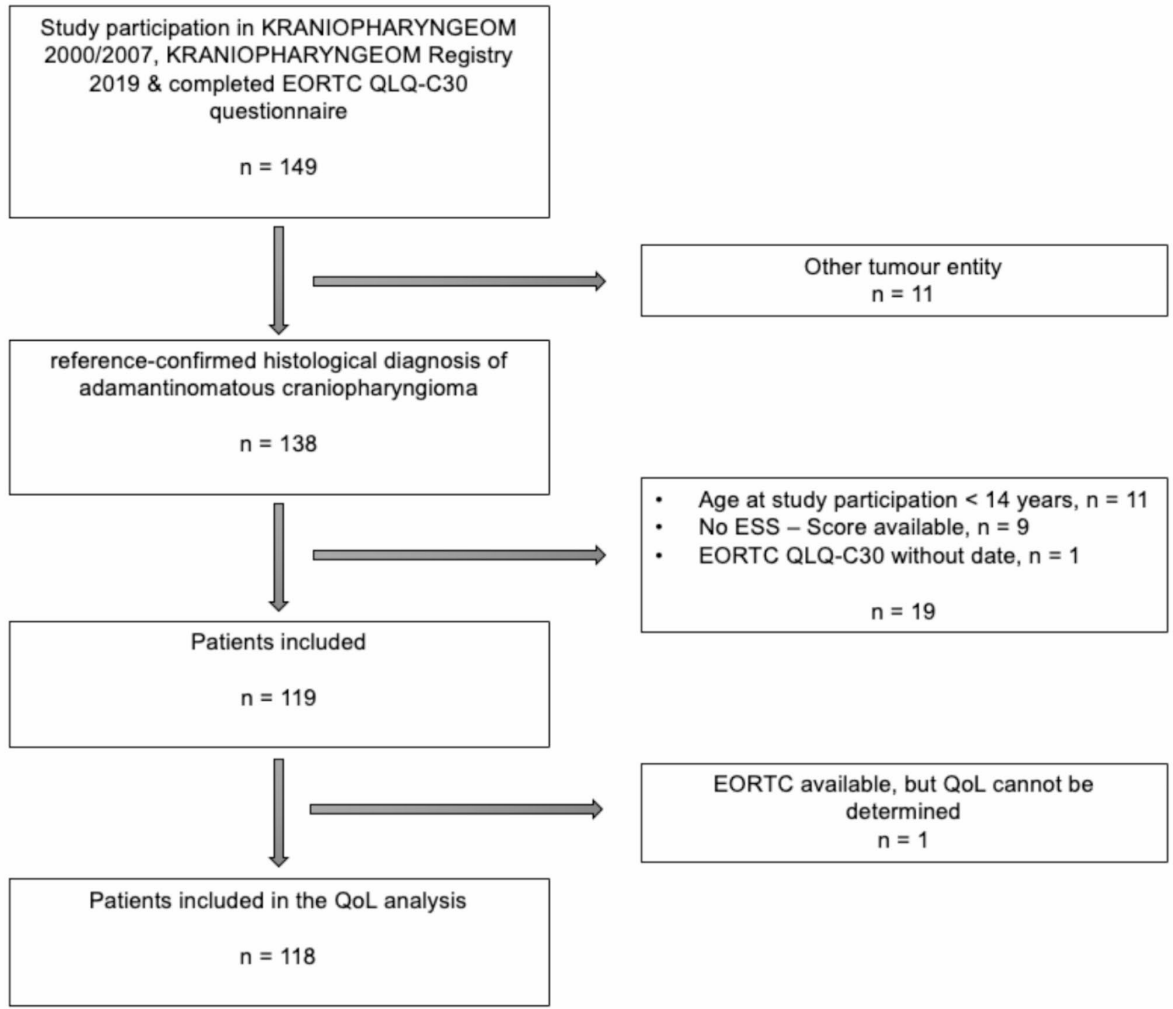
Flowchart of the study cohort of patients with childhood-onset, adamantinomatous craniopharyngioma recruited in the trials KRANIOPHARYNGEOM 2000, KRANIOPHARYNGEOM 2007 and KRANIOPHARYNGEOM Registry 2019. Abbreviations: QoL, quality of life.

After assessment of the eligibility criteria, 119 CP patients (63 female and 56 male) were analysed as part of a cross-sectional study within the KRANIOPHARYNGEOM 2000 (NCT00258453), KRANIOPHARYNGEOM 2007 (NCT01272622) and KRANIOPHARYNGEOM Registry 2019 (NCT04158284) studies. Inclusion criteria were as follows: age at diagnosis < 18 years, age at study participation ≥ 14 years, a reference-confirmed histological diagnosis of adamantinomatous CP, availability of at least one completed EORTC QLQ-C30 questionnaire and one completed ESS questionnaire, written informed consent of the patient or his/her legal representative(s) to participate in the KRANIOPHARYNGEOM 2000/2007/2019 Registry as well as in outpatient follow-up care (Fig. 1). The data collection period was between January 01st, 2000 and December 31st, 2022.

### Instruments

Within the multicentre studies KRANIOPHARYNGEOM 2000, KRANIOPHARYNGEOM 2007 and the KRANIOPHARYNGEOM Registry 2019, the German versions of the EORTC QLQ-C30 (European Organisation for Research and Treatment of Cancer Quality of Life Questionnaire-Core 30, Version 3), and the Epworth Sleepiness Scale (ESS) were completed by the study participants at least at one time point during follow-up. In those cases, where the questionnaires were not completed at the same time, care was taken to ensure that no more than three months elapsed between completion of both questionnaires. All questionnaires were completed by the patients themselves or in assistance of the patients’ legal guardians.

*The EORTC QLQ-C30 version 3.0* is a 30-item questionnaire for which the first 28 items are scored on a 4-point Likert scale. This part includes five functional scales (emotional, cognitive, physical, social, and role functioning), three symptom scales (fatigue, nausea/vomiting, and pain), single items assessing symptoms commonly associated with cancer (dyspnea, insomnia, constipation and diarrhea, loss of appetite), and one item to assess perceived disease and treatment-related financial impact. The last two items (items 29 and 30) form the global health/QoL scale and are scored on a 7-point Likert scale^35^. The questionnaire was analysed according to the official scoring manual^36^. Higher scores in functioning scales indicate better functioning, higher symptom scores indicate higher symptom burden. The higher the QoL-overall score, the better the QoL. In a paediatric cohort, the use of the EORTC QLQ-C30 questionnaire is recommended for patients with an age ≥ 14 years. For younger children, other, more appropriate QoL instruments are recommended.

*The Epworth Sleepiness Scale (ESS)* measures self-perceived daytime sleepiness by using eight items related to falling asleep in typical daytime situations^37^. For each of these situations, participants indicate how likely they are to fall asleep on a four-point scale (0 = I would never fall asleep, 1 = low, 2 = medium or 3 = high probability of nodding off). For the child version, the wording has been slightly changed and one item is adapted to better reflect their daily life (how likely are you to falling asleep at school during lessons instead of falling asleep in a car, while stopped for a few minutes in the traffic). For analyses, we used the widely recommended cut-off score of > 10, indicating abnormal daytime sleepiness^38^.

### Neuroradiological assessment

In accordance with the KRANIOPHARYNGEOM 2007 and KRANIOPHARYNGEOM Registry 2019 protocols, cranial magnetic resonance imaging (MRI) was performed at the time of CP diagnosis and prospectively at 3-months intervals during the first year of follow-up after CP diagnosis. Pre- and post-operative sagittal, coronal and axial MRI images were evaluated by a neuroradiologist, blinded for clinical details, who classified the tumour according to the degree of hypothalamic involvement/surgical lesions: grade 0, no hypothalamic involvement/lesion; grade 1, hypothalamic involvement/lesion of the anterior hypothalamus not involving the mammillary bodies and the hypothalamic area beyond mammillary bodies; and grade 2, hypothalamic involvement/lesion of the anterior and posterior hypothalamic area, i.e. involving the mammillary bodies and the area beyond mammillary bodies^39,40^.

### Clinical assessment

The following data was collected from the registry data: age at diagnosis, age at study, follow-up interval, gender, body height, body mass index (BMI), grade of presurgical hypothalamic involvement, grade of surgical hypothalamic lesions, endocrine deficiencies (central diabetes insipidus/arginine-vasopressin deficiency, hypothyroidism, hypocortisolism, hypogonadism), degree of surgical resection, surgical approach, irradiation, radiation dose and radiotherapeutic technique. The body mass index (BMI = w/h2; w = weight in kilograms, h = height in metres) was calculated as a standard deviation score (SDS) according to the age-related references of Rolland-Cachera et al.^41^. Height was calculated as a standard deviation score (SDS) according to the age-related references published by Prader et al.^42^.

### Statistical methods

Statistical analyses were performed using the SPSS programme from IBM (version 29) and R version 4.2.3. Descriptive statistics such as absolute and relative frequencies or median and interquartile range were used to characterise the study population. As the data was not normally distributed, non-parametric tests such as the Mann-Whitney U test and Spearman correlation were used. Fisher’s exact test and Pearson chi-square test were used for group comparisons of categorical variables.

As this was an observational study, multivariable linear regression models were used in addition to univariable analyses to determine adjusted estimators. A step-wise backwards approach was used for variable selection, with global QOL as dependent variable and age, gender and ESS as independent variables.

A two-sided p-value of ≤ 0.05 was considered as statistically significant. No adjustments were applied for multiple testing. Therefore, the statistics are intended to be exploratory (hypothesis-generating) rather than confirmatory and were interpreted accordingly. In the case of missing values, an imputation procedure was used for QoL variables in accordance with the specifications of the EORTC QLQ-C30 Scoring Manual. If at least half of the items assigned to the respective score were present, the average of these was calculated in order to enable an equivalent calculation of the scores.

## Results

The patient cohort consisted of 119 CP patients, of whom 63 (52.9%) were female and 56 (47.1%) male. Demographic and clinical data are shown in Table 1.

**Table 1:** Characteristics of patients with childhood-onset, adamantinomatous craniopharyngioma recruited in KRANIOPHARYNGEOM 2000, KRANIOPHARYNGEOM 2007 and KRANIOPHARYNGEOM Registry 2019. The p values given refer to the comparison between patients with increased daytime sleepiness (ESS 11–24) and patients with normal scores (ESS 0–10).

Patient characteristics	CP patients	ESS 11–24	ESS 0–10	*p*
Number of patients, n (%)	119 (100)	34 (29)	85 (71)	
Gender (female/male), n (%)	63/56 (53/47)	23/11 (68/32)	40/45 (47/53)	0.033
Age (years) at diagnosis, median (range)	12 (2–17)	13 (7–16)	11 (2–17)	0.042
Age (years) at study, median (range)	22 (14–42)	23 (16–42)	21 (21–37)	0.213
Follow-up interval (years), median (range)	10 (1–39)	11 (2–34)	10 (1–39)	0.883
BMI (SDS) at study, Median (range)^41^	+ 0.82 (-3.23 to + 10.72)	+ 0.53 (-3.23 to + 9.90)	+ 0.91 (-3.09 to + 10.72)	0.887
Missing, n (%)	1 (2)	0 (0)	1 (1)	
Body height (SDS) at study, median (range)^42^	-0.90 (-4.57 to + 3.41)	-0.94 (-3.34 to + 2.90)	-0.88 (-4.57 to + 3.41)	0.902
Missing, n (%)	2 (2)	0 (0)	2 (6)	
Irradiation (XRT), n (%)	53 (45)	15 (44)	38 (45)	0.256
Missing, n (%)	5 (4)	2 (6)	3 (4)	
Irradiation mode				0.689
Proton beam therapy, n (%)	15 (13)	4 (12)	11 (13)	
Photon-based XRT, n (%)	32 (27)	9 (27)	23 (27)	
Other XRT, n (%)	6 (5)	2 (6)	4 (5)	
Unknown, n (%)	5 (4)	3 (9)	2 (6)	
Dosis (Gray), median (range)	54 (10–200)	54 (24–54)	54 (10–200)	0.477
Missing, n (%)	5 (4)	2 (6)	3 (4)	
Degree of surgical resection				0.358
Complete, n (%)	34 (29)	11 (32)	23 (27)	
Incomplete, n (%)	85 (71)	23 (67)	62 (73)	
Surgical access				0.164
Transcranial, n (%)	93 (78)	27 (79)	66 (78)	
Transsphenoidal, n (%)	15 (13)	2 (6)	13 (15)	
Other, n (%)	11 (9)	5 (15)	6 (7)	

ESS, Epworth Sleepiness Scale; XRT, Irradiation.

The median age at the time of initial CP diagnosis was 12 years (range: 2–17 years). At the time of study, the median age of the patients was 22 years (range: 14–42 years). The median follow-up interval between CP diagnosis and conduct of the study was 10 years (range: 1–39 years). The median BMI SDS at the last consultation was + 0.98 SDS for male patients (BMI range: -3.09 to + 8.13 SDS) and + 0.22 SDS for female patients (BMI range: -3.23 to + 10.72 SDS). One patient was excluded from QoL analyses due to missing data in the completed questionnaire, but he was included in the analyses of daytime sleepiness.

Data on presurgical hypothalamic involvement were available for 87 of 119 (73.1%) CP patients. There was no hypothalamic involvement observed in 24 (27.6%) patients (grade 0), while 35 (40.2%) patients presented with grade 1 hypothalamic involvement and 28 (32.2%) patients with grade 2 hypothalamic involvement. Thus, 72.4% of the CP patients, for whom data on presurgical hypothalamic involvement was available presented with grade 1 or 2 hypothalamic involvement.

Data on surgical hypothalamic lesions were available in 84 of 119 (70.6%) CP patients. No postoperative hypothalamic lesions (grade 0) were observed in 6 patients (7.1%). Grade 1 hypothalamic lesions were present in 23 (27.4%) and grade 2 hypothalamic lesions in 55 (65.5%) patients. Accordingly, 92.9% of all patients, for whom reference assessed data on surgical hypothalamic lesions were available, presented with surgical hypothalamic lesions.

For the ESS, we observed 85 patients with a score in the normal range (ESS ≤ 10) and 34 participants with an ESS score > 10, indicating increased daytime sleepiness. Patients with increased daytime sleepiness were not different in terms of BMI SDS when compared to patients with normal scores.

**Fig. 2:**
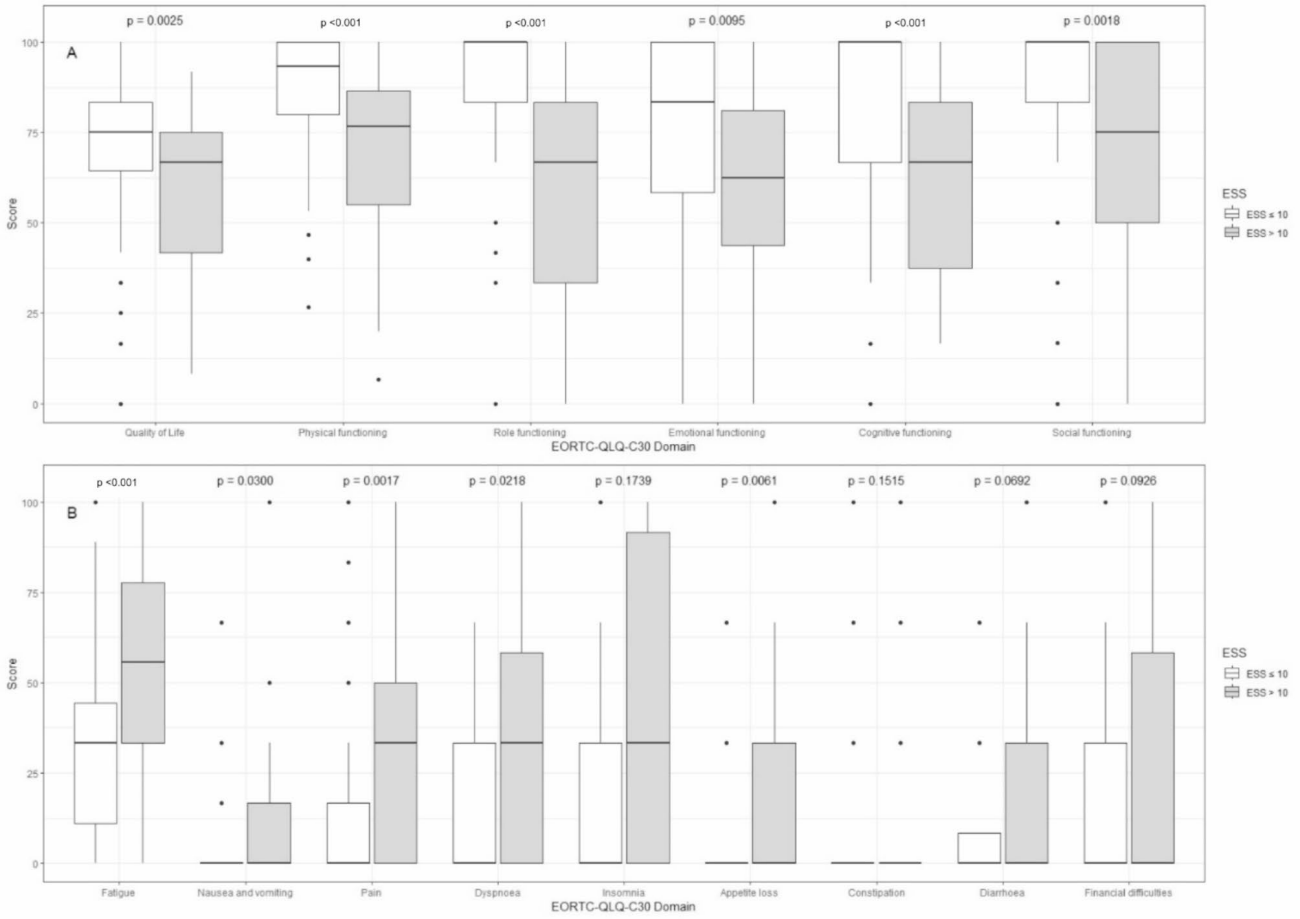
Global quality of life (QoL) and function scales (**A**) and symptoms scales (**B**) of the EORTC QLQ - C30 in relation to the Epworth Sleepiness Scale (ESS). Higher values within the functional scales of the EORTC QLQ-C30 indicate better function and QoL. Global QoL, physical function, role function, emotional and cognitive function and social function were analysed. The boxes represent the interquartile range. The whiskers represent the interquartile range multiplied by 1.5. If the data has already reached its minimum or maximum, the whiskers are only drawn up to this point. However, if there is data behind the antenna, these are displayed as individual points. These data points represent potential outliers. Differences were estimated by Mann-Whitney U test.

In the EORTC QLQ-C30, patients with increased daytime sleepiness achieved significantly lower scores in all functional scales [physical function (*p* < 0.001), role function (*p* < 0.001), emotional (*p* = 0.0095) and cognitive function (*p* < 0.001) and social function (*p* = 0.0018)], and significantly lower scores for global QoL (*p* = 0.0025) (Fig. 2).

They also revealed significantly higher values for the EORTC QLQ-C30 symptom scales [nausea (*p* = 0.030), fatigue (*p* < 0.001), pain (*p* = 0.002) dyspnoea (*p* = 0.022) and loss of appetite (*p* = 0.006)], except for insomnia and obstipation (Fig. 2).

In univariable linear regression analysis, the presence of daytime sleepiness was associated with a decrease in overall QoL by 13.294 (95% CI: -21.91 to -4.68; *p* = 0.003) points when compared to patients without daytime sleepiness (Table 2).

**Table 2:** Multivariable linear regression analysis of quality of life depending on daytime sleepiness (Epworth Score – ESS) and age at study participation in patients with childhood-onset, adamantinomatous craniopharyngioma recruited in the trials KRANIOPHARYNGEOM 2000, KRANIOPHARYNGEOM 2007 and KRANIOPHARYNGEOM registry 2019.

	Unadjusted coefficient	95% confidence interval	*p*	Adjusted coefficient	95% confidence interval	*p*
ESS binary	-13.294	-21.905 to -4.682	0.003	-11.675	-20.182 to -3.167	0.008
Age at study (years)	-1.092	-1.837 to -0.348	0.004	-0.944	-1.677 to -0.211	0.012

After adjustment for age at study, the influence of daytime sleepiness on QoL remained statistically significant (β= -11.675, 95% CI: -20.182 to -3.167; *p* = 0.008). Based on univariable regression, age at study participation had a significant influence on QoL (β = -1.837, 95% CI: -1.837 to -0.348; *p* = 0.004), showing that an increase in age was associated with lower global QoL (Table 2). In the final model with daytime sleepiness, the reduction of QoL associated with higher age at study was smaller compared to the unadjusted model (β= -0.944, 95% CI: -1.837 to -0.348; *p* = 0.004) (Table 2).

Our data did not show a statistically significant association between the grade of presurgical hypothalamic involvement and daytime sleepiness (Table 3).

**Table 3:** Grade of presurgical hypothalamic involvement (HI) and grade of surgical hypothalamic lesions (HL) in relation to the Epworth sleepiness scale (ESS) in patients with childhood-onset, adamantinomatous craniopharyngioma recruited in the trials KRANIOPHARYNGEOM 2000, KRANIOPHARYNGEOM 2007 and KRANIOPHARYNGEOM registry 2019.

Presurgical hypothalamic involvement (HI)^39,40^	ESS 11–24	ESS 0–10	Total	p*
HI grade 0, n (%)	5 (18)	19 (32)	24 (28)	0.238
HI grade 1, n (%)	10 (37)	25 (42)	35 (40)	
HI grade 2, n (%)	12 (45)	16 (26)	28 (32)	
Total, n (%)	27 (100)	60 (100)	87 (100)	
Surgical hypothalamic lesions (HL)^39,40^				
HL grade 0, n (%)	0 (0)	6 (10)	6 (7)	0.042
HL grade 1, n (%)	4 (16)	19 (32)	23 (27)	
HL grade 2, n (%)	21 (84)	34 (58)	55 (66)	
Total, n (%)	25 (100)	59 (100)	84 (100)	

The p values given refer to the comparison between patients with increased daytime sleepiness (ESS 11–24) and patients with normal scores (ESS 0*–*10).

*Differences assessed by Fisher’s exact test.

For surgical hypothalamic lesions in relation to the ESS, Fisher’s exact test yielded a two-sided p-value of 0.042, showing a statistically significant association between the degree of hypothalamic lesions and daytime sleepiness (Table 3). Thus, in contrast to preoperative hypothalamic involvement, the degree of surgical hypothalamic lesions appears to be associated with higher daytime sleepiness.

Nine of 119 patients (8%) were treated with central stimulating agents (3 dexamphetamine and 6 methylphenidate) at the time of study. All patients under medication with central stimulating agents presented with increased daytime sleepiness (ESS > 10). Most patients received endocrine substitution of sex steroids (72%), arginine vasopressin (78%), growth hormone (92%), thyroxine (85%), and hydrocortisone (85%). Differences in terms of endocrine substitution were not observed between the patient subgroups with (ESS 11–24) and without (ESS 0–10) daytime sleepiness (Table 4).

**Table 4:** Medication with central stimulating agents and endocrine substitution in relation to the Epworth sleepiness scale (ESS) in patients with childhood-onset, adamantinomatous craniopharyngioma recruited in the trials KRANIOPHARYNGEOM 2000, KRANIOPHARYNGEOM 2007 and KRANIOPHARYNGEOM registry 2019.

Medication	CP patients	ESS 11–24	ESS 0–10	*p*
Central stimulating medication n (%)	9 (8)	9 (26)	0	
Dexamphetamine, n (%)	3 (3)	3 (9)	0	
Methylphenidate, n (%)	6 (5)	6 (18)	0	
Endocrine deficiencies n (%)				
Hypogonadism	86 (72)	24 (71)	62 (73)	0.937
Arginine vasopressin deficiency	93 (78)	27 (79)	66 (78)	1.000
Growth hormone deficiency	103 (92)	29 (85)	74 (87)	0.351
Hypothyroidism	110 (85)	32 (94)	78 (92)	1.000
Hypocortisolism	101 (85)	32 (94)	69 (81)	0.249

The p values given refer to the comparison between patients with increased daytime sleepiness (ESS 11–24) and patients with normal scores (ESS 0–10).

ESS, Epworth Sleepiness Scale; CP, craniopharyngioma.

## Discussion

It is well known that children with brain tumours are at risk of developing sleep problems because of direct and indirect effects of the tumour and its treatment, in addition to psychosocial and environmental factors^43^. However, these results are in part discussed controversially due to problems of statistical analyses Our study investigated the relationship between daytime sleepiness and health-related QoL in patients with childhood-onset, adamantinomatous CP (≥ 14 years old at study time). Using standardised and validated questionnaires (EORTC QLQ-C30 and ESS), both QoL and daytime sleepiness could be quantified. Regression analyses (Table 2) showed that both, increased daytime sleepiness and older age at study participation were significantly associated with a lower self-assessed QoL. Surgical hypothalamic lesions were associated with increased daytime sleepiness.

The percentage of patients with increased daytime sleepiness in our patient group (29%), was comparable with rates reported in previous studies. Two studies by Müller et al. each investigating a mixed-age group of children, adolescents and adults with childhood-onset CP, observed rates of increased daytime sleepiness of 30% and 35% respectively^12,14^. In a more recent study, Crabtree et al.^44^. found increased daytime sleepiness in 29% of their children and adolescents with CP. The proportion of patients affected by increased daytime sleepiness in these as well as in our study is much higher than found in healthy populations. Studies that were carried out to obtain normative values for the ESS (Sauter et al.^45^, *n* = 239 and more recently, with an even larger sample Sander et al.^38^, *n* = 9711), found that 15.0% and 15.8% of their healthy samples presented with increased daytime sleepiness (see Table 2 in Sander et al.^38^).

The EORTC QLQ-C30 results indicated that patients with increased daytime sleepiness, compared to those with inconspicuous values, suffer from a severe loss of QoL in all functional domains and in global QoL. They also revealed significantly higher scores for most of the EORTC C30 symptoms and symptom scales. It is not yet known and needs to be investigated in further studies, whether and to what extent daytime sleepiness itself contributes to the lower quality of life.

Van Schaik et al. emphasised that sleep disorders are not exclusively associated with a reduced QoL, but also associated with obesity^46^. This association is relevant for patients with hypothalamic obesity. The severity of daytime sleepiness has been shown to be high particularly in CP patients with a BMI > 4 SDS^12^. No differences in BMI SDS between CP patients with increased and those with normal daytime sleepiness were observed in our study.

Older age at the time of participation in our study was significantly associated with lower self-assessed global QoL. In many cases, older age at the time of study participation could be associated with longer follow-up. Our observation on the association between QoL and age of patients with CP, confirms previous findings reported by Beckhaus et al.^25^. The authors discuss that reductions of QoL due to CP diagnosis, might be age-dependent. Older patients could be more aware of their Qol before the CP became symptomatic and thus realize the consequences of impairments leading to a deep and significant decline of Qol measured.

Using EORTC QLQ-C30, Sterkenburg et al. observed reduced physical functioning in patients with hypothalamic involvement of CP. In addition, patients with hypothalamic involvement of CP reached increased scores on the Multidimensional Fatigue Inventory (MFI-20) with regard to physical exhaustion and reduced motivation^24^. Hypothalamic involvement as reported by Sterkenburg et al. was assessed by MRI and/or microscopic inspection during surgery. Hypothalamic involvement was defined as involvement of hypothalamic structures either by tumor growth into the hypothalamus or displacement of hypothalamic structures by the tumour. Due to lower diagnostic quality of imaging in the 1990s, the authors were not able to assess specific grading for hypothalamic presurgical involvement and surgical hypothalamic lesions in their cohort of patients diagnosed with CP before 2001. In our study, we found that surgical hypothalamic lesions and not presurgical hypothalamic involvement were significantly associated with higher ESS and increased daytime sleepiness, showing the influence of surgical hypothalamic lesions on hypothalamic function in terms of circadian rhythms.

Our study has certain strengths and limitations. We assessed whether patients were treated for daytime sleepiness with central stimulating medication. Very few CP patients (8%) took central-stimulating medication at the time of study, which might increase alertness, attention and motor activity. Such pharmaceutical agents could change the results of the ESS questionnaires. However, in spite of such central medication all patients under central stimulation medication presented with still increased ESS > 10 indicating increased daytime sleepiness. Furthermore, it could be speculated that those patients who are particularly affected by daytime sleepiness may be less capable to attend the CP consultations. Given these challenges, those CP patients severely affected by daytime sleepiness may not be adequately represented in this study. Another important aspect is the reliability of self-disclosures about one’s own condition and may particularly affect some neurological patients^47^. In the study by Crabtree et al.^44^ increased daytime sleepiness as indicated by an objective measure (Multiple Sleep Latency Test) was approximately threefold higher than indicated by their modified ESS. Some patients may not recognize or validly report their condition based on self-assessment. Strengths of our study are the use of standardized and validated instruments as EORTC QLQ-C30 questionnaire version 3.0 and ESS and the considerable study size given the rareness of the disease.

Overall, our study provides important insights into the relationship between daytime sleepiness and QoL in patients with CP. For future studies, other factors such as comorbidities, medication use^48^ or lifestyle habits^49^ should be included in the analyses to provide an even more complete picture. Longitudinal studies could be informative in order to determine possible causal relationships. The treatment of daytime sleepiness could thus represent an important strategy for improving QoL, especially in patients who suffer from surgical hypothalamic lesions. Hypothalamus-sparing treatment strategies are recommended for prevention of hypothalamic lesions resulting in increased daytime sleepiness and reduced QoL. Further prospective cohort studies are needed to understand the exact relationships between daytime sleepiness, age, hypothalamic lesions and QoL and to clarify the question of causal relationships^46^.

## Data Availability

The datasets generated during and/or analyzed during the current study are available from the corresponding author on reasonable request.
